# Supplementary material for: Genetic and Clinical Studies of Peripheral Neuropathies with Three Small Heat Shock Protein Gene Variants in Korea
Source: Genes (Basel). 2022 Mar 5;13(3):462. doi: 10.3390/genes13030462 (PMC8949397; doi:10.3390/genes13030462)
Supplement: Supplementary file 1 [file genes-13-00462-s001.zip › genes-1583524-supplementary.pdf]

Table S1 Interpretation of the variants according to the ACMG.

Gene	Family ID	Variant		Interpretation	Conclusion
		Nucleotide	Amino acid		
HSPB1	FC167	c.80G>C	p.R27P	PM2+PP2+PP3	VUS
	FC1005	[c.380G>A] +<br>[c.424T>C]	[p.R127Q] +<br>[p.Y142H]	PM1+PM2+PM5+PP2+PP3+PP4<br>PM1+PM2+PM3+PP2+PP3+PP4	LP<br>LP
	FC371	c.382C>T	p.Q128X	PM1+PM2+PP3	VUS
	FC189<br>FC522,<br>FC567	c.404C>T	p.S135F	PM1+PM2+PM5+PS1+PP1+PP2+PP3+PP4	P
	FC313	c.544C>T	p.P182S	PM1+PM2+ PS1+PS2+PP1+PP2+PP3+PP4+PP5	P
	FC1150	c.560C>T	p.S187L	PM2+PS1+PP2+PP3+PP4+PP5	P
HSPB8	HN104	c.236T>G	p.F79C	PM2+PP3	VUS
	FC585<br>FC1196	c.421A>G	p.K141E	PM1+PM2+PS1+ PS3+PP1+PP3+PP4+PP5	P
	FC031	c.422A>C	p.K141T	PM1+PM2+PM5+ PS1+PS2+PP3+PP4	P
	FC107	c.423G>T	p.K141N	PM1+PM2+ PS1+PS3+PP1+PP3+PP4+PP5	P
HSPB3	FC702	c.352T>C	p.Y118H	PM2+PP1+PP3+PP4	VUS

ACMG, the American College of Medical Genetics and Genomics; LP, likely pathogenic; P, pathogenic; VUS, variant of uncertain significance.

Table S2. Rare uncertain significant variants in three sHSP genes.

Genes	Type	Variants		Mutant allele frequencies[a]			*In silico* analyses[b]			ACMG
		Nucleotide	Amino acid	1000G	gnomAD	KRGDB	PRO	PP2	MUp	
HSPB1	HNPP	c.80G>C	p.R27P	0.0002	0.0002	NR	-3.81*	1.00*	-0.20*	VUS
	CMT1	c.382C>T	p.Q128X	NR	1.1E-05	NR				VUS
HSPB8	HNPP	c.236T>G	p.F79C	NR	NR	NR	-0.55	0.88*	-0.59*	VUS

ACMG, guideline of the American College of Medical Genetics and Genomics; CMT1, Charcot-Marie-Tooth disease type 1; HNPP, hereditary neuropathy with liability to pressure palsies; NR, nonreported; VUS, variant of uncertain significance.

[a] Minor allele frequencies from the 1000 Genomes Project (1000G), the Genome Aggregation Database (gnomAD), and Korean Reference Genome Database (KRGDB).

[b] *In silico* scores of PolyPhen-2 (PP2) ~1, PROVEAN (PRO) <-2.5, and MUpro (MU) <0 indicate pathogenic prediction (* denotes a pathogenic prediction).

Table S3. Phenotypic characterization of IPN patients with mutations in *HSPB1*, *HSPB8*, and *HSPB3* genes.

Patients	Disease type	Sex	Age of exam (yrs)	Age of onset (yrs)	First presenting symptom	FDS	CMTNSv2	Muscle weakness		Muscle atrophy	Sensory loss	Knee/ ankle jerks[c]	Pes cavus
								Arm[a]	Leg[b]				
HSPB1													
FC1005(II-5)	dHMN2B	M	56	45	Gait disturbances	1	4	-	+	Yes	No	D/A	Yes
FC189(IV-2)	dHMN2B	F	58	24	Gait disturbances	4	23	++	+++	Yes	No	A/A	Yes
FC189(IV-4)	dHMN2B	F	51	25	Steppage gait	3	9	++	+++	Yes	No	A/A	Yes
FC189(IV-13)	dHMN2B	F	62	22	Gait disturbance	7	31	+++	+++	Yes	No	A/A	No
FC189(IV-14)	dHMN2B	M	57	24	Lower limb weakness	3	12	++	++	Yes	No	A/A	Yes
FC189(IV-18)	dHMN2B	M	51	19	Paresthesia	3	17	++	+++	Yes	No	A/A	Yes
FC189(V-2)	dHMN2B	F	39	18	Foot drop	3	14	++	+++	Yes	No	A/A	Yes
FC189(V-3)	dHMN2B	M	38	19	Gait disturbances	3	24	+	++	Yes	No	A/A	Yes
FC189(V-6)	dHMN2B	M	26	25	Steppage gait	1	5	+	++	Yes	No	D/A	Yes
FC189(V-10)	dHMN2B	F	34	21	Steppage gait	2	10	+	++	Yes	No	A/A	Yes
FC189(V-11)	dHMN2B	M	33	20	Gait disturbance	1	8	+	+	Yes	No	A/A	No
FC189(V-14)	dHMN2B	M	26	24	Foot drop	1	8	-	+	Yes	No	N/N	Yes
FC522(II-1)	CMT2F	M	35	22	Lower limb weakness	6	22	++	++	Yes	Yes	A/A	Yes
FC567(III-10)	CMT2F	F	64	40	Lower limb weakness	4	23	+++	+++	Yes	Yes	A/A	Yes
FC567(IV-5)	CMT2F	M	42	27	Gait disturbance	1	4	+	++	Yes	Yes	D/A	No
FC567(IV-11)	CMT2F	M	46	18	Lower limb weakness	1	5	+	++	Yes	Yes	A/A	Yes
FC567(IV-13)	CMT2F	M	43	15	Slip down	4	20	+++	+++	Yes	Yes	A/A	Yes
FC313(II-1)	CMT2F	M	40	17	Foot drop	3	17	++	+++	Yes	Yes	N/A	Yes
FC313(III-1)	CMT2F	F	13	11	Gait disturbance	1	5	-	+	No	Yes	N/D	Yes
FC1150(III-1)	CMT2F	F	51	35	Lower limb weakness	1	6	-	+	No	No	N/D	Yes
HSPB8													
FC585(III-5)	dHMN2A	F	34	26	Gait disturbances	4	26	++	+++	Yes	No	A/A	Yes
FC585(III-9)	dHMN2A	F	25	17	Lower limb weakness	2	13	+	++	Yes	No	H/N	Yes
FC585(III-10)	dHMN2A	M	32	19	Lower limb weakness	3	13	+	+++	Yes	No	A/A	Yes

FC585(III-12)	dHMN2A	F	27	18	Lower limbs weakness	2	10	+	+	Yes	No	D/D	Yes
FC1196(II-1)	dHMN2A	M	30	19	Steppage gait	2	10	+	++	Yes	No	A/A	Yes
FC031(II-1)	CMT2L	M	28	13	Gait disturbances	6	21	+++	+++	Yes	Yes	A/A	Yes
FC107(II-2)	dHMN2A	M	45	18	Steppage gait	4	17	++	+++	Yes	No	A/A	Yes
HSPB3													
FC702(III-1)	CMT2	M	57	25	Steppage gait	3	15	+	+++	Yes	Yes	A/A	Yes
FC702(IV-2)	CMT2	F	29	17	Gait disturbances	2	9	+	++	Yes	Yes	A/A	Yes

CMT2, Charcot-Marie-Tooth disease type 2; CMTNSv2, CMT neuropathy score ver. 2; dHMN, distal hereditary motor neuropathy; F, female; FDS, functional disability scale; M, male.

[a] Muscle weakness in upper limbs: + = intrinsic hand weakness 4/5 on medical research council (MRC) scale; ++ = intrinsic hand weakness <4/5 on MRC scale; +++ = proximal weakness; - = no symptom

[b] Muscle weakness in lower limbs: + = ankle dorsiflexion 4/5 on MRC scale; ++ = ankle dorsiflexion <4/5 on MRC scale; +++ = proximal weakness; - = no symptom

[c] Deep tendon reflexes: D = diminished; A = absent; N = normal, H = hyper reflex

Table S4. Electrophysiological values of IPN patients with mutations in *HSPB1*, *HSPB8*, and *HSPB3* genes.

Patient	Age at exam (yrs)	Disease duration (yrs)	Motor nerve conduction								Sensory nerve conduction					
			Median nerve		Ulnar nerve		Peroneal nerve		Tibial nerve		Median nerve		Ulnar nerve		Sural nerve	
			CMAP (mV)	MNCV (m/s)	CMAP (mV)	MNCV (m/s)	CMAP (mV)	MNCV (m/s)	CMAP (mV)	MNCV (m/s)	SNAP (μV)	SNCV (m/s)	SNAP (μV)	SNCV (m/s)	SNAP (μV)	SNCV (m/s)
HSPB1																
FC1005(II-5)	56	11	19.2	61.5	13.9	61.5	0.2	32.8	0.5	41.3	41.3	50.0	19.5	48.0	15.9	42.4
FC189(IV-2)	58	34	A	A	A	A	A	A	A	A	23.1	44.9	21.2	39.8	17.2	38.3
	59	35	A	A	A	A	A	A	1.4	39.3	36.5	44.1	23.6	39.1	17.8	36.0
FC189(IV-4)	50	25	6.8	50.3	7.1	55.0	A	A	A	A	25.7	40.8	17.8	39.0	24.5	34.3
	51	26	7.5	50.3	5.9	53.7	A	A	A	A	32.0	41.1	24.9	40.8	18.2	33.0
FC189(IV-13)	61	39	8.0	45.8	1.1	41.2	A	A	A	A	36.8	41.1	9.4	38.7	14.4	32.6
	62	40	2.8	46.5	A	A	A	A	A	A	32.1	41.6	16.7	37.8	14.8	34.5
FC189(IV-14)	57	33	7.0	56.6	2.1	47.5	A	A	A	A	28.0	41.2	11.9	38.9	19.3	32.6
	65	31	7.9	51.7	0.8	50.8	A	A	A	A	33.4	46.9	19.3	41.4	26.0	32.6
FC189(IV-18)	50	31	8.1	47.8	0.3	42.0	A	A	0.3	25.2	37.6	42.1	11.2	37.5	27.6	32.2
	51	32	2.1	41.6	0.9	41.9	A	A	A	A	23.4	39.6	12.5	39.9	13.9	33.6
FC189(V-2)	38	20	0.1	38.2	A	A	A	A	A	A	43.9	40.5	17.3	40.4	14.3	36.2
FC189(V-3)	36	17	1.4	50.0	2.4	51.0	A	A	A	A	15.5	43.2	12.4	40.1	10.5	37.4
	37	18	1.2	49.1	2.1	50.0	A	A	A	A	30.6	42.8	12.2	39.9	13.1	35.1
FC189(V-6)	25	1	18.6	63.8	13.4	58.3	0.3	43.2	0.1	38.7	37.4	47.3	16.9	41.7	26.6	34.3
FC189(V-10)	35	14	14.6	59.3	12.3	55.8	A	A	0.2	30.8	26.6	41.1	18.6	38.3	10.8	34.7
	36	15	14.8	55.9	12.0	55.6	A	A	0.2	33.5	33.5	41.8	21.1	38.9	13.2	34.3
FC189(V-11)	33	13	19.4	58.7	18.1	50.0	A	A	0.9	42.0	20.5	41.3	10.7	40.5	12.7	32.4
	34	14	20.1	53.8	17.7	52.9	A	A	1.6	43.8	28.5	43.2	13.3	40.5	23.7	34.4
FC189(V-14)	26	2	17.4	59.8	13.6	59.0	A	A	0.7	31.2	26.7	43.5	18.6	40.9	13.6	32.8
	27	3	19.5	61.2	11.3	60.0	A	A	0.6	30.1	33.8	44.9	27.2	41.4	13.4	38.1
FC522(II-1)	34	12	7.7	61.5	4.5	52.1	A	A	0.7	29.5	29.1	46.4	15.6	43.0	19.2	31.3
	38	16	8.8	60.5	2.4	47.5	A	A	1.1	37.1	29.4	52.5	14.2	50.8	13.7	46.7
FC567(III-10)	64	24	3.2	54.5	1.4	59.4	A	A	A	A	38.2	44.9	30.2	43.1	29.8	45.5
FC567(IV-5)	42	15	12.8	60.5	2.2	58.8	1.3	25.1	0.1	24.4	38.6	42.3	18.5	38.6	28.2	30.6

	43	16	15.1	54.6	1.8	49.8	0.2	36.2	A	A	72.5	51.7	26.2	46.7	28.8	42.4
	45	18	14.9	57.3	2.2	47.8	0.2	37.8	A	A	58.0	48.3	23.3	47.2	21.6	46.7
FC567(IV-11)	44	26	14.8	62.9	0.7	57.9	A	A	A	A	24.7	46.9	11.4	40.2	10.6	36.7
	47	29	12.1	51.9	1.3	44.5	A	A	A	A	38.8	51.7	20.6	47.8	10.1	41.9
FC567(IV-13)	42	27	12.2	61.8	0.8	54.2	A	A	A	A	22.4	44.1	18.8	40.3	26.3	37.0
FC313(II-1)	40	33	A	A	0.6	27.3	1.3	33.7	0.5	36.2	12.0	40.0	10.9	40.0	22.8	35.7
FC313(III-1)	13	2	3.3	50.7	10.3	51.5	0.3	49.0	7.8	42.1	27.6	35.8	20.4	44.2	1.3	38.9
	15	4	3.6	48.9	12.9	43.8	0.9	47.2	6.3	41.3	27.4	32.7	21.7	44.3	1.5	40.0
FC1150(III-1)	51	16	20.0	59.5	13.7	62.2	3.9	43.4	3.4	46.0	39.0	56.4	41.1	46.1	5.5	40.0
HSPB8																
FC585(III-5)	34	8	14.2	60.0	17.7	65.7	A	A	A	A	54.5	54.3	27.6	52.4	23.5	48.3
FC585(III-9)	24	7	16.9	57.4	22.6	62.1	5.2	48.4	2.8	40.9	50.5	46.7	26.4	49.1	36.1	41.2
FC585(III-10)	32	13	10.3	49.8	17.0	59.6	A	A	A	A	20.9	37.6	26.5	48.5	18.1	36.8
FC585(III-12)	27	9	18.3	64.0	19.4	71.4	6.5	50.1	0.5	41.0	36.9	52.0	58.8	62.8	34.6	40.3
FC1196(II-1)	30	11	18.7	63.4	15.0	58.7	A	A	A	A	40.7	52.0	20.6	48.0	20.0	43.8
FC031(II-1)	15	2	7.2	46.9	4.7	52.4	A	A	A	A	3.2	32.2	6.0	47.0	A	A
	26	13	4.4	46.2	5.3	55.3	A	A	A	A	A	A	A	A	A	A
	27	14	4.7	45.3	5.2	54.5	A	A	A	A	A	A	A	A	A	A
FC107(II-2)	44	16	15.0	57.7	3.4	50.0	A	A	A	A	48.6	48.5	36.7	45.6	31.3	43.8
HSPB3																
FC702(IV-2)	27	2	20.0	53.3	13.5	56.7	0.8	42.7	0.5	39.6	4.4	44.3	8.7	40.7	A	A
	29	12	22.9	49.6	19.3	52.9	2.5	32.3	0.6	32.9	8.4	40.0	7.2	37.9	A	A

A, absent action potential; CMAP, compound muscle action potential; MNCV, motor nerve conduction velocity; SNAP, sensory nerve action potential; SNCV, sensory nerve conduction velocity. Normal NCVs: motor median nerve, ≥50.5 m/s; ulnar nerve, ≥51.1 m/s; peroneal nerve, ≥41.2 m/s; tibial nerve, ≥41.1 m/s; sensory median nerve, ≥39.3 m/s; ulnar nerve, ≥37.5 m/s; sural nerve, ≥32.1 m/s. Normal amplitudes: motor median nerve ≥6 mV; ulnar nerve, ≥8 mV; peroneal nerve, ≥1.6 mV; tibial nerve, ≥6 mV; sensory median nerve, ≥8.8 µV; ulnar nerve, ≥7.9 µV; sural nerve, ≥6.0 µV.
